# Engaging children, adolescents and young adults living with rare diseases as research partners: practical considerations for meaningful involvement

**DOI:** 10.3389/fped.2026.1886270

**Published:** 2026-09-29

**Authors:** Maria Cavaller-Belaubi, Elena-Alexandra Tataru, Donato Bonifazi, Daniel J. O’Connor, Francis P. Crawley, Michelle A. Farrar, Segolene Gaillard, Ainara Gómez Puigvert, Diana Kwast-Hoekstra, Louise Kearney, Samantha Parker, Sabina Schmidt Goldstein-Becerra, Karsten Vanden Wynagert, Anneliene H. Jonker

**Affiliations:** 1 EURORDIS-Rare Diseases Europe, Paris, France; 2 International Rare Diseases Research Consortium (IRDiRC), Paris, France; 3 Fondation Maladies Rares, Paris, France; 4 TEDDY- European Network of Excellence for Paediatric Clinical Research, Bari, Italy; 5 The Association of the British Pharmaceutical Industry (ABPI), London, United Kingdom; 6 Good Clinical Practice Alliance (GCPA) Europe, Bruxelles, Belgium; 7 Department of Neurology, Sydney Children’s Hospitals Network, Sydney, NSW, Australia; 8 Discipline of Paediatrics and Child Health, School of Clinical Medicine, UNSW Medicine and Health, UNSW Sydney, Sydney, NSW, Australia; 9 Neurosciences Research Australia, Sydney, NSW, Australia; 10 CIC Inserm, Kids France, CHU-Lyon, Lyon, France; 11 Laboratoire de Biométrie et Biologie Évolutive, Université Lyon 1, Villeurbanne, France; 12 YPAG Kids Barcelona, Barcelona, Spain; 13 Department of Geriatric Medicine and Department of Epidemiology, University Medical Center Groningen, University of Groningen, Groningen, Netherlands; 14 Stichting (on)beperkt Vooruit!, Almere, Netherlands; 15 Fortrea, Kingston Upon Thames, United Kingdom; 16 Italfarmaco SpA, Milan, Italy; 17 The International Children’s Advisory Network, Los Angeles, CA, United States; 18 Ghent University Hospital, Ghent, Belgium; 19 Duchenne Parent Project, Veenendaal, Netherlands; 20 HTSR, TechMed Centre, University of Twente, Enschede, Netherlands

**Keywords:** children, patient engagement, rare diseases, research and development, therapy development, young people

## Abstract

Engagement of patients has become increasingly recognized as an important principle in medical and health research and development. However, the engagement of young people affected by a rare disease- including children, adolescents, and young adults - has lacked consistent practical guidance that is structured, developmentally appropriate, and ethically grounded. Without clearer practical considerations, researchers may have limited guidance to effectively include this crucial population in ways that respect their evolving autonomy, accommodate diverse contexts, and ensure meaningful impact. This is especially important in rare disease research, where the majority of affected people are children. To address this gap, the International Rare Diseases Research Consortium convened a Working Group on Young People's Engagement, comprising 16 international experts. The group included two young people alongside academic and industry researchers, patient advocates, and clinical specialists with experience working directly with youth. The Working Group reviewed core requirements for engaging young people in research and examined case studies illustrating diverse engagement approaches. Drawing on existing patient engagement frameworks, the group developed expert-informed practical guidance, including a checklist, to support researchers in considering appropriate engagement strategies across developmental stages and research contexts. Strengthening the engagement of young people in rare disease research may help ensure that different types of research, including innovation development, shaping study design, outcome measures and policy decisions, are meaningful, relevant, and reflective of their lived experiences.

## 1 Introduction

Patient engagement is defined as the active, meaningful collaboration of patients, their representatives, relatives, caregivers, or advocates with academic and industrial researchers, healthcare professionals and knowledge brokers, across all stages of the drug development and delivery lifecycle, healthcare innovation strategies and policy making (1, 2). In recent years, it has become increasingly recognized as an important principle to ensure that healthcare innovations and research output are patient-centered and provide answers to real-world needs (3).

Translating the principles of patient engagement into clinical research has yielded measurable benefits. In clinical trials, meaningful patient engagement has been associated with improved recruitment and retention rates, as well as helping to ensure that the outcomes assessed are relevant and meaningful to patients and reflect their lived experience (4, 5). In addition, engagement can inform study design, participant-facing materials, and endpoint selection, helping to ensure that research is both feasible and appropriate for the population being studied. This may reduce the risk of avoidable challenges arising from study procedures, outcomes, or assumptions that do not adequately reflect the perspectives and needs of participants (4, 6). This may be particularly important when conducting research involving children, adolescents, and young adults, whose needs and priorities may differ from those assumed by adult researchers and clinicians. Most importantly, patient engagement helps ensure that medicines and other health innovations are developed with consideration of the real-world needs, preferences, and priorities of those living with the disease (7).

In rare disease research, patient engagement is a complex process that serves as a catalyst for innovation, helping to address many of the methodological and practical challenges inherent in studying small, heterogeneous patient populations (8). This engagement is especially crucial in rare disease research, where traditional research methods often struggle to fully capture the lived experiences of those affected. Although individual rare diseases affect no more than 1 in 2,000 people, collectively they impact over 350 million individuals worldwide (9, 10). The rarity of individual conditions, together with their clinical heterogeneity, geographically dispersed patient populations, and limited evidence base, creates significant challenges for research and can hinder effective patient engagement (11–13).

Many rare diseases are diagnosed in childhood, with early engagement typically mediated by parents or caregivers (9, 14). As children advance in age, the young persons could start to be involved in decision-making progressively shifting toward shared input and, ultimately, patient autonomy, requiring careful integration of both voices to ensure ethical and representative research. Recognition and support of this evolving dynamic, from parent-led input to shared decision-making and ultimately patient autonomy, are essential to conducting ethically sound research that reflects lived experience and patient needs (15, 16). At the same time, many pediatric-onset diseases are progressive, meaning that young people may face increasing physical limitations and loss of independence precisely when they move toward adulthood. Engagement approaches must therefore accommodate this dual trajectory. Similarly, conditions diagnosed in young adulthood require flexible strategies that balance emerging independence with continued parental involvement (17). Meaningful engagement during adolescence can also support a smoother transition from pediatric to adult care and strengthen the development of self-management skills.

Despite increasing emphasis on engaging children, adolescents and young adults in rare disease research, defined as young people throughout the paper, their active involvement remains limited and inconsistently applied. This gap is especially concerning given the distinct developmental and psychosocial challenges faced by young people, which influence their experiences, and decision-making, and can ultimately shape both research outcomes and care strategies (18–20). Furthermore, Article 12 of the United Nations Convention on the Rights of the Child states that children have a right to express their views, to have them heard and for their views to be given due weight in all matters that affect them (21). However, despite this right, young people are still not routinely involved in healthcare decision-making despite this right. Although some initiatives demonstrate successful engagement of young people (see case studies included in Table 1), variability in methods, evaluation, and impact measurement persists.

**Table 1:** Spectrum of youth engagement.

Level	Description	Role of young people
Inform	One way communication where participants receive updates	Young people receiving study information
Consult	Researchers seek opinions or feedback	Surveys, conversations with youth representatives within patient associations
Advise	Participants contribute lived experience and suggestions	Youth advisory boards
Co-produce	Collaborative shaping of research questions, design and approach	Young people are co-designing protocols and materials
Shared decision-making	Active involvement in steering decisions, reviewing materials, refining processes	Young people are part of the governance committees i.e., research prioritisation committees, patient-centred outcome selection committees, regulatory consultation workshops
Youth-led	Participants act as co-researchers or lead elements in the study	Young people initiate or conduct research i.e., funding decisions

Continuum of young people engagement in research. Adapted from the IAP2 Spectrum of Public Participation, the table illustrates different levels of youth involvement in research, from being informed and consulted through to advisory roles, co-production, participation in decision-making, and youth-led research. The table has been adapted to reflect the evolving autonomy and unique contributions of young people in health and rare disease research. The choice among these modes of engagement should be guided by the research question, context, feasibility.

The need to incorporate the voice of young people is further illustrated by work from the Endo-ERN Transition of Care Working Group. In a survey of 80 centers across 21 European countries, substantial variability was identified in transition practices, including limited psychological support, inconsistent use of structured transition protocols, and gaps in continuity of care. These findings highlight the challenges of developing services for adolescents and young adults without a comprehensive understanding of their experiences and priorities. The study reinforces the importance of directly engaging young people in healthcare and research design to ensure that systems intended to support autonomy, adherence, and long-term outcomes reflect the needs of those most affected (22).

When adolescent and young people's perspectives are absent, care and research risk being poorly aligned with their needs. This challenge is compounded by the lack of practical guidance that is structured, developmentally appropriate, and ethically grounded for engaging young people in rare disease research. This is limiting researchers' ability to support evolving autonomy, adapt to diverse contexts, and ensure that participation meaningfully shapes research and outcomes (15). To help address this gap, the International Rare Diseases Research Consortium (IRDiRC) Working Group on Young People Engagement conducted an initiative, documented as an expert-informed perspective drawing on selected literature, working-group expertise and experience, and illustrative case examples. This included a landscape scan of current engagement practices, a review of case studies to identify best practices and challenges and the development of a checklist providing practical support.

This paper, as such, brings together key findings, considerations, and lessons learned to provide developmentally sensitive, and ethically informed guidance for engaging young people in research. It also offers practical resources that may support researchers working with young people in adapting engagement approaches across different contexts and stages of research and clinical care.

## 2 Methodology

Sixteen experts participated in the study, including professionals working with young people and two young people themselves, all with experience in rare diseases. Participants were recruited through an open online call, which was disseminated widely through multiple channels, including social media platforms (LinkedIn), the IRDiRC newsletter and website, Research Alliance (ERDERA) network, and various patient organizations, and young people networks. Selection criteria were established to ensure that participants had relevant experience of working with young people with rare diseases or, in the case of young participants, lived experience themselves. Particular attention was also paid to achieving broad geographical representation and diversity. Professional members were selected to ensure a wide range of expertise, including representatives from industry, patient advocacy organizations, research institutions, and clinical practice. The young people were from Greece and Spain, respectively, and brought complementary perspectives based on their lived experiences. One of the participants was a member of a Young Persons Advisory Group (YPAG) network, the other one is a member of a Greek young patient's association. Together, they represented different rare disease communities. In addition, five patient advocates participated in the Working Group, contributing broader perspectives from the rare disease community. To respect privacy, information regarding the specific conditions they represented was not collected. A total of 10 online meetings were conducted, each addressing predefined topics identified through group brainstorming, using the Nominal Group Technique to facilitate structured discussion and consensus building. Most members participated throughout the entire process. To ensure continuity and sustained engagement, Working Group members were expected to attend a minimum of seven of the ten meetings. Draft recommendations and accompanying figures were prepared by the group chairs, subsequently discussed and refined by the full panel, and circulated to ten youth organizations (TEDDY KIDS Network (KIDS ANDO, KIDS Bari, EYE YPAG London, KIDS Rare Hellas, KIDS Albania and KIDS Umbria, KIDS Kosova, POLPEDNET Warsaw, RAiN Ireland, PEG's Invents) for additional feedback on relevance, feasibility, approaches, inclusivity and meaningful participation. Young people also indicated which parts of the checklist were considered most important. Finally, illustrative cases contributed by participants were discussed collectively, and the group reached consensus on the key lessons and practical insights that could be drawn from each case to inform the recommendations.

## 3 Engagement of young people in research

Key engagement activities of young people are diverse and span the different phases of research and development including: identifying research priorities, assisting in study design, developing study materials, conducting research, data analysis and interpretation, and dissemination and knowledge translation (23–25). Research engagement can be viewed as a continuum ranging from informing and consulting participants through to collaboration and stakeholder-led research, with increasing influence over research decisions and conduct (Table 1).

In pediatric research, integrating the perspectives of young people and their caregivers through participatory approaches can improve communication, motivation, participant adherence and the ethical conduct of clinical research. Co-developed consent and assent processes may further enhance recruitment, participation, and representation, while supporting data sharing, especially relevant in rare diseases (26–28). Engagement activities should be transparently reported, including their aims, methods and impact using established guidance (29). Several frameworks outline principles for meaningful youth engagement, including the PARTNER principles, which is a globally developed framework to support structured patient partnership in research, development and healthcare decision-making; PARTNER promotes participatory and developmentally appropriate approaches that integrate young people's perspectives throughout research. It supports accessible co-design using inclusive, age-appropriate methods; rights-based ethics that ensure informed assent and consent; and team-centered support that safeguards young people's well-being while respecting their independent voices (30). PARTNER principles provide an important framework for supporting long-term networks with youth organizations, building research capacity among young people and professionals, and ensuring recognition and accountability through ongoing evaluation and feedback. This paper extends this framework by drawing on real-world case studies and practical tools to illustrate how youth engagement can be adapted across different research settings, developmental stages, and levels of involvement.

## 4 Examples of engagement of young people

At global level, several initiatives have sought to operationalize young people's engagement in research and contribute to the harmonization of engagement approaches across settings. To inform the development of practical guidance, we examined a series of case studies representing diverse examples of youth engagement from the different working group members. Table 2 provides a comparative overview of these initiatives including their setting, purpose, engagement methodology, target age group, accessibility safeguards and key lessons learned.

**Table 2:** Overview of global examples of engagement of young people, including the names of the initiatives, challenges encountered and lessons learned.

Name of initiative	eYPAGnet, Europe	Medical research council, Zimbabwe	Fortrea, US	TEDDY network, Europe	DMD voice
Phase of engagement	Throughout the research lifecycle.	Transition from pediatric to adult care (over 12 years).	Ethics and consent in early-stage research	Clinical research	Study design and qualitative data collection
Challenges and opportunities	Creating consistent structures across countries; balancing young people's input with research timelines; adapting to each research project; meaningful PPI activity impacting projects and young people.	Teens shared their hesitancy toward adult clinics due to attachment with pediatric themes, fear of being misunderstood, lack of adolescent-centered spaces, language used by clinicians, fear of losing free medications, reluctance to be grouped with older adults and negative health outcomes.	Communicating clinical trial information to young people effectively.	Hearing what young people think, making learning tools for young people, and helping them take part in international projects.	Obtaining meaningful, ethically sound qualitative data from younger participants with varying cognitive and emotional readiness
Lessons learned	Structured, long-term engagement frameworks and partnerships with existing young people’ networks enhance participation and impact.	Acknowledging transition-related anxieties; need for structured engagement; establishment of dedicated adolescent clinics and joint preparatory workshops with pediatric and adult teams; importance of listening to caregivers to reinforce compassionate communication.	Co-designing materials with young people, using visual aids, young people-friendly language, and digital tools improves understanding and reduces fear; engaging young people directly leads to more effective and ethical consent processes.	Developing informational materials together with young people and giving them a voice in international conferences and debates.	Successful engagement of young participants requires intentional, age-appropriate methods, specialized interviewer training, and close collaboration with caregivers and patient organizations.

Practices identified as examples of good practice informed the development of the checklist described in this manuscript.

### 4.1 Case study: eYPAGnet - involving young patients and parents in the design of clinical trials

eYPAGnet is a European network of experts in Patient and Public Involvement (PPI), embedded in children's hospitals and clinical research facilities, working directly with young patients, parents and patient organizations. eYPAGnet mission is to ensure meaningful involvement of young people across all stages of Health research. eYPAGnet develops bespoke PPI plans adapted to each project, identifying what activities are relevant at each stage (design, conduct, dissemination), which participants should be involved, and which methodology should be used. Every plan includes an Impact Assessment to evaluate effects on both research and participants and is aligned with best practice standards and governance, including reimbursement policies and confidentiality agreements.

One example involved a multi-country European platform trial in a rare disease, where eYPAGnet coordinated focus groups with families in France, Spain and the UK (31). The PPI plan included a review of the study design and acceptability of the innovative design. The involvement led to concrete protocol changes such as adapted visit schedules, improved information for assent/consent, and the inclusion of patient-reported outcomes. Country-specific feedback also shaped decisions on follow-up locations and check-up frequency.

Through this model, eYPAGnet shows how a bespoke well-designed PPI plan can directly influence trial design in pediatric clinical research.

### 4.2 Case study: ethical and regulatory challenges in pediatric clinical trials in Zimbabwe

Clinical trials in children are vital due to physiological differences from adults, and for African children, such research ensures drug efficacy and safety in local contexts. However, in Zimbabwe, the absence of a dedicated legal framework for pediatric research presents significant ethical and practical challenges. Informed consent for children is typically obtained from parents or legal guardians, guided by international ethical codes and local laws. Zimbabwe relies on two acts: the Medicines and Allied Substances Control Act (32) and the Anatomical Donations and Post-Mortem Examinations Act (33). While both require guardian consent, they lack explicit provisions for pediatric research, child assent, or considerations of the child's best interest.

This gap became evident during a collaborative HIV clinical trial involving the UK's Medical Research Council and research centers in Zimbabwe and Uganda, which aimed to use the potential of the African youth to transform health research in Africa (34). Researchers struggled to obtain consent for orphaned children whose caregivers, though culturally recognized as guardians, lacked legal authority under Zimbabwean law. Formal guardian appointment is a complex legal process, often impractical in community settings. In response, local stakeholders have worked toward procedures that align international standards with Zimbabwe's legal and cultural context (e.g., context-specific assent approaches, documentation of best interests, and routes for recognizing de facto caregivers), highlighting the need for explicit, child-centered provisions in national guidance.

### 4.3 Case study: redesigning pediatric assent forms with the child's voice in mind

A second case study comes from a contract research organization, Fortrea, in the United States (35). Recognizing the child's right to be heard, Fortrea undertook a redesign of its pediatric assent forms to make them more understandable and engaging for young clinical trial participants (36). In collaboration with the International Children's Advisory Network (iCAN) (37), Fortrea introduced cartoon illustrations and simplified language to better communicate trial procedures to children.

A cross-functional team reviewed the original assent templates and created new versions incorporating child-friendly visuals and feedback from both children and caregivers. Feedback from iCAN participants indicated that visuals and plain language improved comprehension and reduced anxiety, provided images were age-appropriate and avoided unnecessary realism that could provoke fear. Children also proposed assistive features (e.g., read-aloud options, plain-language glossaries and short animations) to support diverse literacy and accessibility needs.

These findings led to revised assent templates tailored to different age groups, aligning with European guidelines (38). This work underscores the importance of involving young people in the research design process not just as participants, but as contributors ensuring that their voices shape the tools meant to inform and protect them. Importantly, the working group noted that chronological age alone should not determine the format or content of engagement and assent materials. Rather, materials should be adapted to the young person's cognitive and developmental maturity, communication needs, disease context, and individual circumstances.

### 4.4 Case study: promoting children's participation in health policy: the role of TEDDY KIDS network in the council of Europe

TEDDY promotes children's rights in pediatric research by actively involving young people through the TEDDY KIDS Network, which connects Young Persons Advisory Groups (YPAGs) across Europe to foster participation, empowerment, and equal access to healthcare. A notable example of meaningful engagement is TEDDY KIDS Network's collaboration with the Council of Europe (CoE) in developing the 2023–2024, guidance document on “Children's Participation in Decisions about Their Health”, aimed at supporting healthcare professionals in implementing child-centered practices across Europe (39). Members of the TEDDY network, including KIDS Bari and KIDS Albania, contributed directly to ensure the guide reflected children's real needs and experiences.

In addition, a CoE video, “Have Your Say!” was co-designed by TEDDY KIDS members to raise awareness among children about their right to health, within the framework of the Council of Europe Strategic Action Plan on Human Rights and Technologies in Biomedicine (2020–2025) (40). From 2 to 4 April 2025, the CoE organized an international conference in Strasbourg on the European Strategy for the Rights of the Child, bringing together young delegates, TEDDY KIDS, experts, and activists to discuss children's health rights, sex education, and the protection of Ukrainian children's rights. These initiatives highlight how continuous engagement of young people shapes policy, creates educational tools, and strengthens their direct involvement with institutions, demonstrating their capacity to influence international research standards and defend their rights in healthcare and research.

### 4.5 Case study: qualitative interviews with Duchenne patients and caregivers (DMD voice)

Incorporating the perspectives of caregivers and patients is essential for a comprehensive understanding of lived experience, particularly in chronic and rare conditions such as Duchenne muscular dystrophy (DMD), though it raises methodological and ethical challenges. The DMD Voice study (NCT06925269), an ongoing multinational qualitative interview study in the US, Canada, Spain, and Italy, illustrates both the value and complexity of engaging younger participants (41). The study explores how boys aged 12 years and older with DMD and their caregivers perceive functional ability, independence, and quality of life through remote semi-structured interviews.

The interview guide was adapted to be developmentally appropriate and sensitive to the emotional impact of discussing disease progression, using shorter, concrete questions and everyday examples. Younger participants often struggled with open-ended questions and abstract concepts such as independence, further complicated by varying levels of disease awareness and understanding of prognosis. This case highlights the importance of intentional methodological design when including younger participants in qualitative research. While young participants offer unique insights into daily life and adaptation, meaningful and ethical engagement requires age-appropriate materials, trained interviewers, and early involvement of caregivers and patient advocacy groups. Consulting caregivers about participants' understanding of their condition can also inform analysis and help balance ethical considerations with data richness, ensuring young voices are neither excluded nor tokenized.

## 5 When and how to engage with young people

Despite progress across individual initiatives, there remains a need for practical resources that can help researchers, sponsors, patient organizations and other stakeholders consider how best to engage young people in rare disease research. In response to this need, the IRDiRC working group developed a complementary tool intended to support planning, reflection, and discussion around youth engagement. This tool draws on existing evidence, published frameworks, and real-world case studies to offer considerations that may be adapted to different research settings.

### 5.1 Checklist

Engaging young people meaningfully in research requires a structured, inclusive, and ethically grounded approach. To guide when and how to involve young people and clarify expected benefits, a checklist was developed to support structured, age- and context-sensitive participation in line with WHO classifications. In rare diseases context, where the pediatric voice can be involved at different development milestones, from priority-setting and protocol/materials development to feasibility/pilots, data interpretation and dissemination. Where direct pediatric engagement is not feasible, proxy input (parents/carers/siblings/young people advocates) will be needed. In such cases, proxy perspectives should be considered as complementary sources of information, while acknowledging that they may not fully capture the individual experiences, preferences, and priorities of the young person themselves. The Practical Considerations for Engaging Young People in Research (Figure 1) provides a concise framework to ensure such involvement is intentional, respectful and impactful. This checklist emphasizes the importance of clarifying the initiative's purpose, ensuring alignment with the priorities and lived experiences of young people and establishing a clear identity for the engagement. It also highlights the need to embed inclusion and equity from the outset, particularly by reaching underserved groups, treating youth as equal contributors and ensuring fair, preferred forms of compensation. It calls for co-design with young people by tailoring communication and where practical settings and formats are adapted to their needs such as aspects like reasonable session length (60–90 min) with breaks, timing that avoids school hours, and transport/data support foster accessibility and comfort.

**Figure 1:**
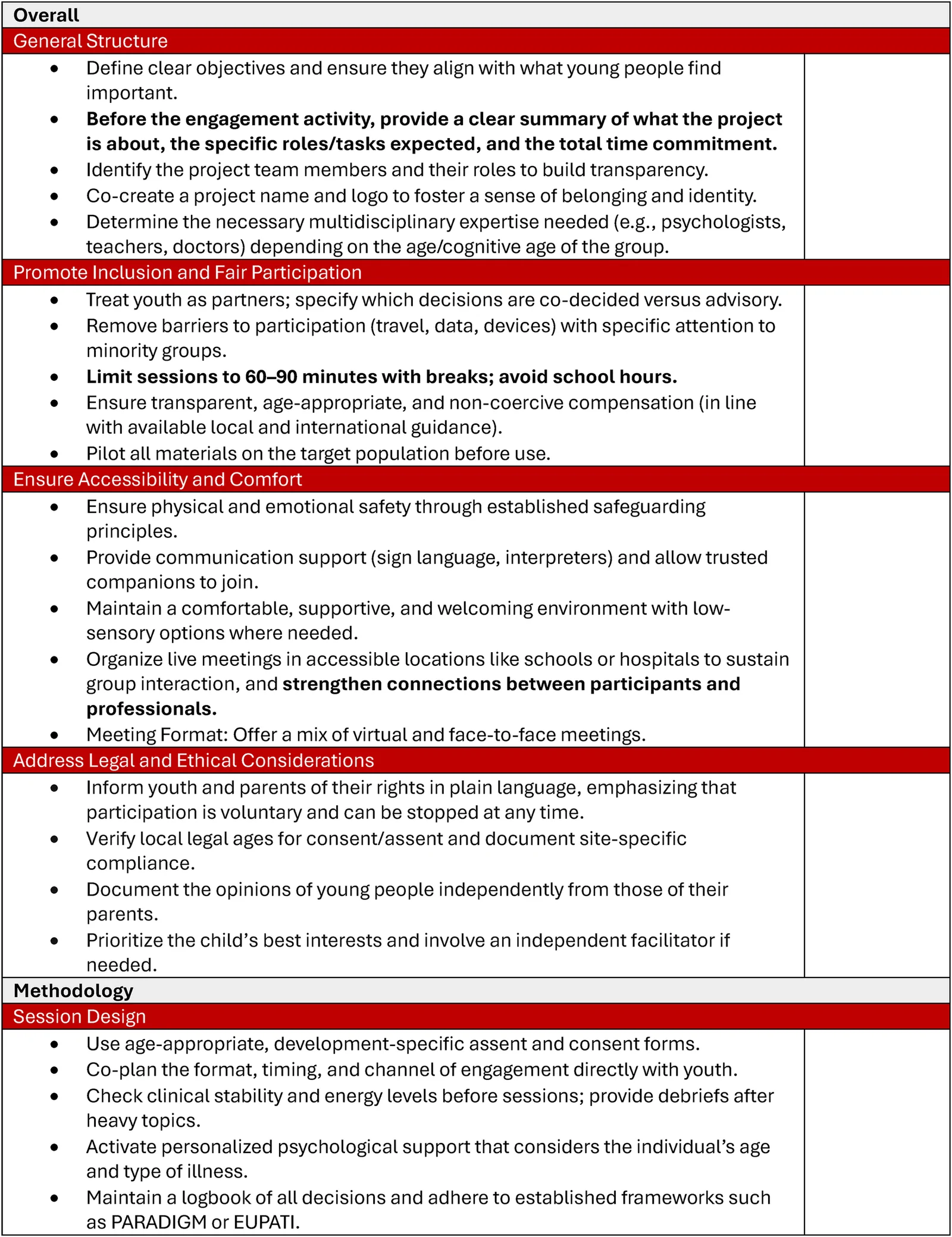
Continue

**Figure 1:**
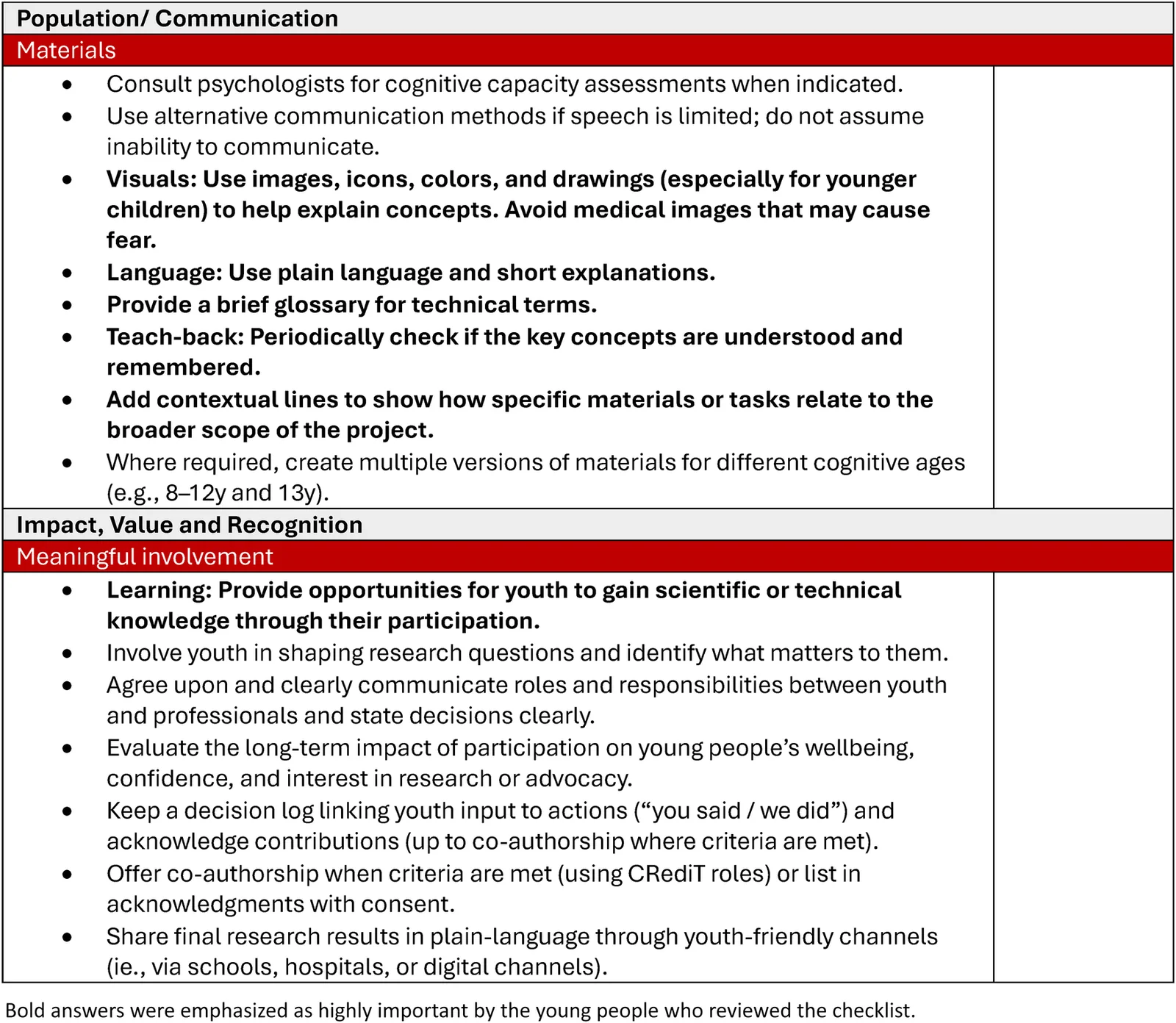
(Continued).

Methodologically, the checklist underscores ethical and compliance across jurisdictions, including age-appropriate assent in addition to parental/guardian consent and respect for global variations in decision-making capacity. Finally, recognition in scientific outputs (up to co-authorship where criteria are met) and lay-language reporting help validate young participants as co-creators in research and to document how youth input has informed decisions.

## 6 Limitations

This study has several limitations. First, the recommendations were developed through an expert consensus process and therefore reflect the perspectives and experiences of the participating group; although informed by the available literature, they do not represent the results of formal evidence grading or systematic guideline development process. Second, participants were recruited through an open call and youth networks, which may have introduced selection bias, as individuals with a particular interest or experience in rare diseases and youth engagement may have been more likely to participate. Third, while input from young people and youth organizations was incorporated, the number and diversity of perspectives represented may not fully capture the experiences of all young people living with rare diseases across different conditions, cultures, and healthcare systems. Finally, the developed recommendations and decision-making tools require further evaluation through implementation studies and external validation to assess their usability, acceptability, and impact in real-world settings.

## 7 Conclusions

Building meaningful engagement with young people in rare disease research requires embedding their perspectives, values, and lived experiences at every stage of the research process. Recognizing that young people are not a monolithic group but a diverse population with varying needs and capacities, researchers must adopt flexible, responsive strategies grounded in both ethics and practical realities. The creation of practical considerations may help bridge the current implementation gap, providing guidance that can be adapted across research settings, cultural contexts, and stages of development.

The tools and practical considerations developed within the IRDIRC Young People Engagement working group are not static: they will evolve in response to changing needs of young people, new methods and emerging ethical challenges. Applying and refining these practices, could assist the research community to foster genuine partnerships with young people, strengthen research outcomes, and contribute to more inclusive and responsive health innovation in rare diseases.

Importantly, engagement with young people should be viewed as complementary to, rather than a replacement for, engagement with other key stakeholders, including caregivers, clinicians, researchers, industry, regulators, and policy-makers. Bringing these perspectives together can support more holistic decision-making and help ensure that research reflects both the scientific and clinical realities of rare diseases and the priorities of those living with them.

## Data Availability

The original contributions presented in the study are included in the article, further inquiries can be directed to the corresponding author.
